# Characteristics of breast cancers detected by screening mammography in Taiwan: a single institute’s experience

**DOI:** 10.1186/s12905-023-02445-6

**Published:** 2023-06-21

**Authors:** Hsin-Ju Han, Yuan-Chia Chu, Jane Wang, Yi-Chen Lai, Ling-Ming Tseng, Chi-Cheng Huang

**Affiliations:** 1 Department of Surgery, Taipei Veterans General Hospital, Taipei, Taiwan; 2 Information Management Office, Taipei Veterans General Hospital, Taipei, Taiwan; 3 Big Data Center, Taipei Veterans General Hospital, Taipei, Taiwan; 4 Department of Information Management, National Taipei University of Nursing and Health Sciences, Taipei, Taiwan; 5 Department of Radiology, Taipei Veterans General Hospital, Taipei, Taiwan; 6 Department of Radiology, National Taiwan University College of Medicine Taipei Veterans General Hospital, Taipei, Taiwan; 7 Department of Nurse-Midwifery and Women Health, Taipei, Taiwan; 8 Department of Nursing, College of Nursing, National Taipei University of Nursing and Health Sciences, Taipei, Taiwan; 9 Comprehensive Breast Health Center, Taipei Veterans General Hospital, Taipei, Taiwan; 10 School of Medicine, National Yang Ming Chiao Tung University, Hsinchu, Taiwan; 11 Institute of Epidemiology and Preventive Medicine, National Taiwan University, Taipei, Taiwan

**Keywords:** Breast cancer, Screening, Mammography, Interval breast cancer, Taiwan, Symptomatology

## Abstract

**Background/aim:**

Breast cancer is the most common female malignancy in the world. Nearly ninety percent of screening-detected breast cancers were diagnosed with earlier stages of 0 to II in Taiwan. It’s widely acknowledged that mammography screening of breast cancer can achieve the goal of early diagnosis and treatment in terms of preventive task while neglected interval cancers are associated with unfavorable tumor characteristics and worse outcomes. The purpose of this study was to explore the characteristics of screening-detected breast cancers in Taiwan.

**Materials and methods:**

Both screening and diagnostic mammography examinations with accompanied BI-RADS (breast imaging-reporting and data system) classification were extracted from the health information system and linked to cancer registry in Taiwan. Enrolled population included those attending their first mammography between 2012 and 2016, excluding subjects with previous breast cancer, or with missing or incomplete data. We compared treatment outcomes between breast cancers with either initial screening or diagnostic mammography (control group), and investigated the compositions of breast cancers detected by screening mammography through direct chart reviews.

**Results:**

A total of 84,246 screening and 61,230 diagnostic mammography sessions were performed from 2010 to 2020. More positive results (BI-RADS 0, 3, 4 and 5) were observed for those attending the first diagnostic than the first screening mammography (43.58% versus 16.12%, *p* < 0.001). Earlier stages (0 and I) distribution (92% versus 81%, *p* < 0.0001), better survivorship (overall survival: 96.91% versus 92.17%, *p* = 0.007) and a lower HER2 (human epidermal growth factor receptor II) positive status and lower tumor grade were noted in breast cancers with initial screening rather than diagnostic mammography. Among 26,103 mammography screening invitees between 2012 and 2016, 325 breast cancers were ascertained from cancer registry. Of these, 234 had one, 72 had two and 19 had three episodes of mammography before cancer diagnosis. Extensive chart reviews revealed that women with and without breast symptoms constituted 29.9 and 70.1% of the 325 screening-detected breast cancers, with the latter further divided into false negative results (interval cancer), diagnosed at the first mammography, diagnostic at the secondary or subsequent mammography and those with a delayed biopsy or confirmatory imaging constituted (5.2, 47, 10.5 and 7.4%).

**Conclusion:**

Screening-detected breast cancers were a mixture of women with and without symptoms, those with a false negative result, true negative results with cancer detected at subsequent mammography and non-adherers. Despite this, efficacy of mammography screening was ascertained in Taiwan from this study. To further enhance earlier detection and decrease false negativity, the impact of repeated mammography, and additional sonography for symptomatic women, compliance following a positive screening mammography should not be overemphasized.

**Supplementary Information:**

The online version contains supplementary material available at 10.1186/s12905-023-02445-6.

## Introduction

Breast cancer is the most common female malignancy in the world and has become one of the leading causes of death in women [1, 2]. In Taiwan, breast cancer ranks the first for cancer incidence and the fourth for cancer death in women. The peak age of breast cancer diagnosis falls between 45 and 69 years old. Therefore, screening program in Taiwan subsidizes women aged 45–69 and women aged 40–44 years who have a family history of breast cancer with biennial mammography since 2009, and approximately 860,000 women receive this service, with more than 4,000 breast cancers identified each year. It is widely acknowledged that better survival is related to earlier diagnosed stages. The 5-year overall survival of stages 0 to II is approximately ninety percent in breast cancers.

Biennial mammography can reduce breast cancer mortality by 41% and reduce the incidence of advanced breast cancer by 30% [3]. The reduction in breast cancer mortality is due to screening itself and advances in multidisciplinary treatments [4]. Many countries have promoted mammography screening programs for decades [5]. However, the harms and benefits of mammography screening should be deciphered thoroughly. The main merit is the reduction of women dying from breast cancer and a shift toward earlier diagnosed stages. In contrast, overdiagnosis, false positive results and interval breast cancer (IBC) are potential harms for mammography screening, which should be informed to invitees [6–8]. One study conducted in Taiwan indicated that mammography has reached the level of recommendations from the American College of Radiology (ACR) regarding recall rate and positive predictive value, with the exception for sensitivity [9]. In Taiwan, both mobile (out-reach) and hospital-based screening services are widely available, while the effectiveness of hospital-based (in-reach) mammography screening is still under debate [10–12]. The objective of this study was to evaluate the performance of mammography screening from a single institute, reflecting the actual circumstance within a tertiary referral medical center by reviewing medical records of diagnosed breast cancers, with the possibility to find improved methods.

## Materials and methods

### Data collection and study population

This study extracted mammography examinations with BI-RADS (breast imaging-reporting and data system) classification from 2010 to 2020 at the Taipei Veterans General Hospital (VGH-TPE). The targeted population were women attending their first mammography at the VGH-TPE from 2012 to 2016. Patients had at least two years of medical record antecedent to their first mammography and five years of follow-up were enrolled while women who had previous mammography from 2010 to 2011 were excluded. The population were divided into two groups; patients who visited our outpatient department with a diagnostic mammography implemented by the National Health Insurance (NHI) Administration were the control group, while those attending the screening mammography service at the expense of the Bureau of Health Promotion constituted the screening group. Diagnostic and screening mammography examinations were supported by the NHI and Tobacco Tax and the Tobacco Health Welfare Surcharge from the Bureau of Health Promotion, respectively.

**Fig. 1:**
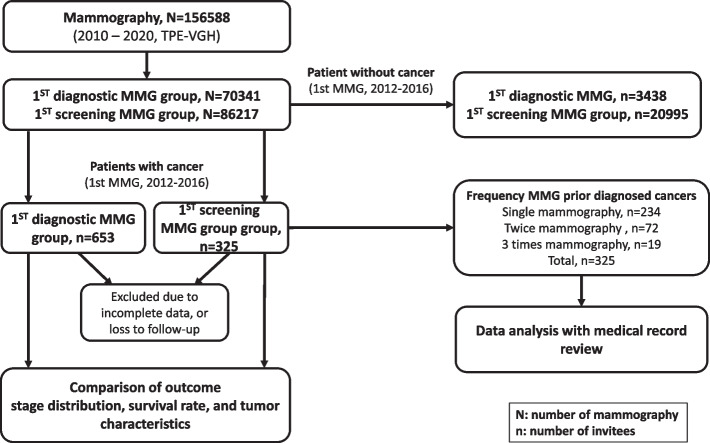
Over view of mammography episodes and diagram of study population, 2010–2020

All data were extracted from the health information system and linked to the cancer registry through a unique ID; both in situ (stage 0) and invasive lesions (stage I-IV) were considered as breast cancer index cases. We compared treatment outcomes of breast cancer patients diagnosed from these two groups. We also conducted chart reviews for breast cancers whose first round of mammography was screening-tailored (Fig. 1). Parameters such as recall rate, cancer detection rate, positive predictive value, sensitivity, specificity, false positive, false negative rate were reported. The frequency and interval from mammography to cancer diagnosis, and symptomatology at the time of screening mammography were collected through chart review (screening group only). Patients with a previous history of breast cancer as well as those with missing, incomplete data or loss to follow-up were excluded. The whole study protocol was reviewed and approved by IRB with informed consent waived.

### Definition of recall rate, delayed examination, interval cancer, and scenarios of screening-detected breast cancers

Recall rate was defined as the percentage of positive screening results (BI-RADS Category 0, 3, 4, or 5) divided by all screened subjects. Those who failed to attend subsequent confirmatory examinations as recommended by the corresponding BI-RADS classifications (every two years for both BI-RADS 1 and 2 and every 6–12 months for BI-RADS 3, while the full workup for BI-RADS 4 and 5 should not exceed 6 months) were classified as having a delayed subsequent imaging for patients’ sake. Interval cancers referred to breast cancers diagnosed within two years after a negative screening (BI-RADS 1 and 2) or those with a cancer diagnosed more than 6 months after a false-positive screening (BI-RADS 0, 3, 4 and 5 and subsequent negative imaging or biopsy results). In addition, screening-detected breast cancers were divided into scenarios such as symptomatic and asymptomatic cased documented from medical records at the time of screening, as well as those diagnosed from the first or subsequent rounds of mammography examinations. Based on contemporary guidelines, the findings of mass, architectural distortion, focal asymmetry and microcalcification were further evaluated with sonography and/or spot compression/magnification view of mammography.

### Prognostic comparison

In our study, a small tumor (< 2 cm, i.e. pTis and pT1) without regional lymph node invasion (N0) was defined as an earlier stage (stage 0-I). Pathology reports with estrogen receptor (ER)/progesterone receptor (PR) positivity, ductal carcinoma in situ (DCIS) and mucinous carcinoma were defined as factors of better prognostication.

### Statistical analysis

All data were analyzed using the SAS software (SAS Institute Inc., Cary, NC) with Student’s t test, log-rank test, and Cox’s regression adopted appropriately. All statistical tests were two-sided, and an α-level of 0.05 was considered statistically significant.

## Results

### BI-RADS distributions of women with their first screening and diagnostic mammography

A total of 145,476 mammography examinations were conducted between 2010 and 2020, with 61,230 being diagnostic and 84,246 being screening mammography (Table 1). Healthy individuals without a previous history of breast cancer attending their first diagnostic mammography from 2012 to 2016 had more positive results (BI-RADS 0, 3–5) than those with the first screening mammography (43.58% versus 16.12%, χ2-test: *p* < 0.001, Table 2).

**Table 1:** Mammography imaging examinations at the VGH-TPE during 2010 to 2020

Imaging modality	The first mammography		
	Diagnostic	Screening	Total
Mammography, bilateral	53358	0	53358
Mammography, spot view	7872	0	7872
Mammography screening in 40–44 years	0	570	570
Mammography screening in 45–69 years	0	83676	83676
Total	61230	84246	145476

**Table 2:** BI-RADS distributions for women with the first mammography during 2012 to 2016 without a final diagnosis of cancer

BI-RADS	The first mammography				
	Diagnostic		Screening		Total
0	877	25.14%	2207	10.51%	3084
1	78	2.24%	2519	12%	2597
2	1793	51.4%	15028	71.58%	16821
3	340	9.75%	1042	4.96%	1382
4	255	7.31%	117	0.56%	372
5	48	1.38%	19	0.09%	67
6	41	1.18%	2	0.01%	43
missing	56	1.61%	61	0.29%	117
Total	3488		20995		24483

### Characteristics of breast cancers from women with the first screening and diagnostic mammography

**Fig. 2:**
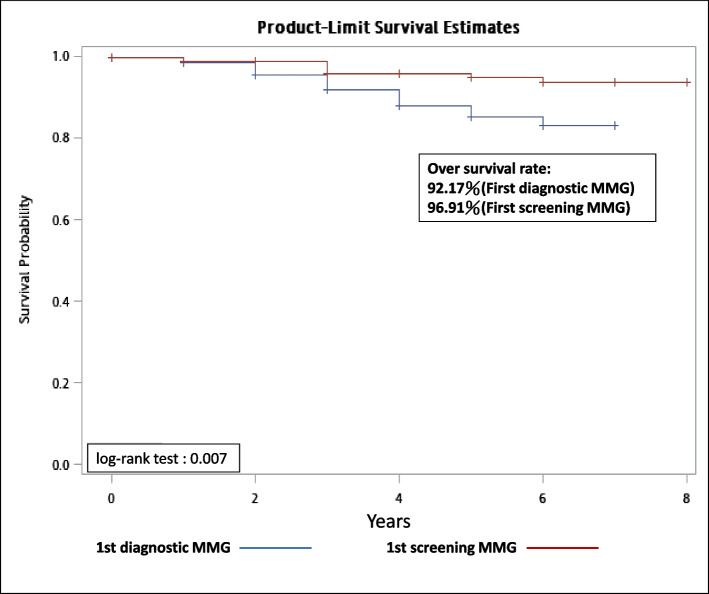
Overall survival between breast cancers with initial diagnostic and screening mammography

Tumors from women with the initial screening mammography displayed a significantly lower human epidermal growth factor receptor II (HER2)-positive status (*p* = 0.0006) and tumor grade (*p* = 0.012) than those with the initial diagnostic mammography, which was not the case for ER and PR (*p* = 0.2521 and 0.0826, respectively). Earlier stage distributions (0-I stage) were observed for breast cancers diagnosed from screening than diagnostic mammography (92% versus 81%, χ2- test: *p* < 0.0001), as well as better overall survival (96.91% versus 92.17%, log-rank test: *p* = 0.007 (Fig. 2, Table 3 and Supplement Table 1).

**Table 3:** Breast cancer characteristics of (a) estrogen receptor (ER), (b) progesterone (PR), (c) human epidermal growth factor receptor II (HER2), (d) grade, and (e) pathological stage distributions between women with initial screening/diagnostic mammography

(a)						
The first mammography	**ER (%)**					
	Negative			Positive		Total
Diagnostic	11 (18.6)			516 (81.4)		634
Screening	45 (15.5)			245 (84.5)		290
Total	163			761		924
(b)						
The first mammography	**PR (%)**					
	Negative			Positive		Total
Diagnostic	175 (27.7)			456 (72.3)		631
Screening	65 (22.3)			226 (77.7)		291
Total	240			682		922
(c)						
The first mammography	**HER2 (%)**					
	Negative			Equivocal	Positive	Total
Diagnostic	470 (74.8)			5 (0.8)	153 (24.4)	628
Screening	213 (74.2)			13 (4.5)	61 (21.3)	287
Total	683			18	214	915
(d)						
The first mammography	**Grade (%)**					
	I	II	III	Total		
Diagnostic	47 (7.6)	413 (66.7)	159 (25.7)	619		
Screening	33 (11.5)	204 (71.1)	50 (17.4)	287		
Total	80	617	209	906		
(e)						
Pathological stage	The first mammography					
	Diagnostic	Screening		Total		
0	84 (14.26)	73 (25.7)		157		
I	195 (33.1)	117 (41.19)		312		
II	196 (33.2)	71 (25)		267		
III	78 (13.2)	19 (6.69)		97		
IV	36 (6.11)	4 (0.14)		40		
Total	589	284		873		

### Efficacy of hospital-based (in-reach) screening mammography

From 2012 to 2016, 26,103 women had their initial screening mammography; among them, 325 breast cancers were ascertained from cancer registry. Of these, 234, 72, and 19 underwent mammography once, twice and thrice before cancer diagnosis. The overall recall rate was 20.34%, resulting in a cancer detection rate of 1.26%, positive predictive value of 5.29%, false negative rate of 0.235%, sensitivity of 84.9%, specificity of 80.5% (Supplement Table 2).

### Scenarios of breast cancers from women with the initial screening mammography

**Fig. 3:**
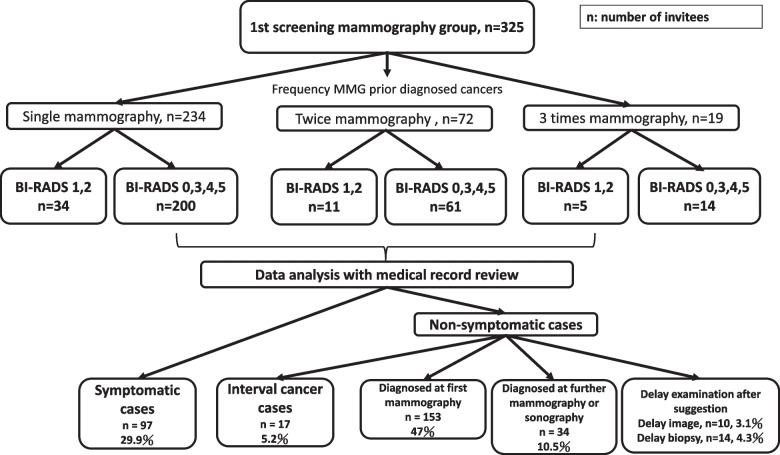
Flow chart for symptomatic and asymptomatic breast cancers with initial screening mammography from 2012 to 2016 (*n* = 325)

From chart reviews of 325 screening-detected breast cancer index cancers, the proportion of symptomatic and asymptomatic cases were 29.9% and 70.1. Among asymptomatic women at the initial screening, interval cancers, diagnosed from the first screening, diagnosed from subsequent mammography and cases with a delayed biopsy or confirmatory imaging were 5.2, 47, 10.5, 4.3 and 3.1%, respectively (Fig. 3, Supplement Tables 3–5). Among breast cancers diagnosed from subsequent mammography, 34 patients were as ascertained with a true negative result from their initial screening mammography by two independent radiologists (JW and YCL).

### Scenarios of interval breast cancers

There were 17 interval cancers, with 7 and 10 being false-positive and false-negative from the initial screening mammography. Among them, 11 patients had positive results from both sonography and diagnostic mammography following a negative screening result, 5 had positive results from sonography only (diagnostic mammography not performed), and one patient had positive sonography but negative diagnostic mammography. Only 4 patient (23.5%) were with better pathology types (one DCIS, two mucinous carcinoma and one invasive lobular carcinoma) and 10 patients (59%) were hormone receptor (ER or PR) positive. Compared to 34 breast cancers diagnosed at subsequent mammography (true negative result of initial screening), 19 (56%) were with better pathology types and 27 patients (79%) were hormone receptor positive (Supplement Table 6).

## Discussion

The implement of mammography screening has reduced the mortality from breast cancer worldwide. Population-based screening has also improved breast cancer treatment outcomes by early detection. However, the effectiveness of hospital-based screening (in-reach) program remains undetermined. In our study, the recall rate was much higher among women with initial diagnostic than screening mammography. it’s quite intuitive that women with suspicious symptoms preferred ambulatory care rather than screening for a timely examination and diagnosis, and we believe that participants of diagnostic mammography were with more breast symptoms, and consequently, higher recall rate and cancer detection rate, which were evidenced in our study, indicating fewer symptoms experienced by women utilizing mammography for the purpose of screening.

The recall rate of patients with and without a cancer detection was 20.34 and 16.12% among women with initial screening mammography, both were higher than that from a population-based Taiwanese study between 2004 and 2012 (around 9.3–10.0%, ref. [9]). Symptomatic cases were underestimated from the NHI administration data analysis. A higher recall rate may result in unnecessary procedures, additional medical costs, and compromise invitees’ psychological status and health behavior [7]. On the other hand, recalled subjects may become more adhered to follow-up and therefore have a better chance to be diagnosed with earlier cancer stages. It deserves notice that participants of hospital-based screening might have a different baseline risk of breast cancer than those attending community-based screening programs such as services delivered by a mobile car.

The cancer detection rate in this study was 1.26%, which was much lower than previous domestic data. The low yield of cancer detection might be attributed to the widespread of breast awareness and education, and more cancers had been diagnosed from outpatient department, rather than mammography screening. Indeed, highly accessible medical resources in Taiwan may lower the need for mass screening, and some healthy women might take advantage of outpatient clinic rather than screening program for their breast checkup. This retrospective study reviewed medical records to separate symptomatic from asymptomatic breast cancers from screening. The former included patients with symptoms, such as breast mass, pain or bleeding from nipple, and the latter includes patients without symptom. This method could help us clarify true clinical scenarios of screening program. Our data found that symptomatic invitees might sum up to 29.9% of women with initial screening, which means that the cancer detection rate might be even lower if only asymptomatic women were enrolled. Despite the heterogeneous nature of screening invitees as a mixture of both asymptomatic/symptomatic women, the efficacy of mammography is still ascertained from our study. Moreover, elimination of false negative results through repeated mammography surveillance as well as enhance the compliance of screened positive cases could further augment modern screening policy, as evidenced from the study.

One meta-analysis argued that the number needed to invite was lower for mammographic screening program without clinical breast examination than that with, and both screening methods provided higher benefits for women aged 50 years and over [13]. In Taiwan, the high social economic status enhance women’s breast awareness and they tend to seek medical advice even without a prominent breast symptom. Besides, the high coverage of NHI eliminate the barrier of breast imaging access, consequently lowering the cancer detection rate from screening. In current study, targeted population focused on women aged 45–69 and women aged 40–44 years with a family history, while all participants resided in urban regions. Whether different screening populations in developing countries benefit more from mammography with or without a clinical breast examination needs further evaluation.

A recent European study reported that more unfavorable tumor characteristics were noted in interval than screen-detected breast cancers [14]. In current study, we reviewed medical records and found that more breast cancers with better pathological types and hormone receptor positive status were detected from scheduled subsequent mammography than interval cancers. Likewise, our data also identified worse clinical characteristics of women with a IBC. With the rate of 6.5 per 10,000 screening episodes, the incidence of Taiwanese IBC was much lower than that from the European study, ranged from 8.4 to 21.3 per 10,000 screenings [14]. Women with breast symptoms have an increased risk of IBC than those without, and it was estimated that IBC risk increased by 3.9 per 10,000 screenings with a clinical breast lump [15]. Therefore, separation of symptomatic from asymptomatic women during screening not only reflects the true efficacy of mammography, but also decreases the incidence of IBC.

In our study, all 17 interval cancers had positive sonography findings before cancer diagnosis, but only 11 had positive confirmatory mammography results, and one case even had a benign confirmatory mammography report. The other 5 cancers completed confirmatory mammography after the cancer diagnosed and approved through sonography-guided biopsy. Actually, all interval cancers were diagnosed by sonography-guided needle biopsy. It hinted that interval cancers tended to display sonography-detectable lesions, and it was quite straightforward to take advantage of readily available sonography-guided procedure in a timely fashion. A recent review had suggested that supplemental sonography could detect occult breast cancers missed by mammography. In addition, sonography has been suggested as a means of supplementary tool augmenting mammography screening in women with dense breasts [16]. Another Japanese study reported that mammography screening alone demonstrated low sensitivity, whereas adjunctive sonography was associated with increased sensitivity [17]. Hence, sonography should be added to screening programs under specific conditions, such as unpalpable masses with benign nodular lesions in mammography or dense/extremely dense breasts, as well as young age population. In Asia, the prevalence of dense breast and young female breast cancer is higher than Western counterpart, consequently we hypothesize that additional sonography might be of great value to lower the incidence of IBC for Taiwanese women, especially for symptomatic invitees.

Furthermore, it had been reported that the reduction in delayed confirmatory imaging with/without an accompanied biopsy may help improve breast cancer survival. In our study, the proportion of women with a delay in subsequent confirmation was 7.3%. This value was higher than that of 4.8% from a European study, which decreased gradually from 1997 to 2016 [18, 19]. Establishing a well-organized system for the management of recalled women from screening mammography is crucial in improving breast cancer outcomes.

Some studies have shown that breast cancers who were non-receipt of screening mammography was associated with late-stage disease and mortality as well as poor tumor characteristics, suggesting that mammography screening may improve breast cancer outcomes [20, 21]. Other studies also pointed out that consecutive mammography screening can improve breast cancer mortality [22, 23]. Our analysis also ascertained that breast cancers diagnosed from mammography screening had a better overall survival, more favorable tumor characteristics and earlier stage distributions than those diagnosed clinically with an initial diagnostic mammography. Future researches should focus on the impact of screening frequency in breast cancer detection with a larger sample size.

This study had some limitations. As mentioned previously, a large proportion of symptomatic invitees were found in women with their first screening mammography, which was inevitable from the hospital-based in-reach setting of current study. Despite this, our data still showed better outcomes of breast cancers after initial screening than diagnostic mammography. Additionally, this study did not compare different screening interval, while a previous study reported that universal biennial mammography was the most effective strategy for early detection of breast cancer compared to risk-based screening and clinical breast examination [3]. Lastly, all data were collected from one medical center, which did not represent all institutes providing screening service, and the results might be different in other areas.

## Conclusion

Our study ascertained the feasibility of using administration data and chart review for breast cancers to evaluate the effectiveness of mammography screening from a tertiary medical center in Taiwan. Despite screening invitees including both asymptomatic and symptomatic women, mortality reduction still augmented the efficacy of mammography screening from a hospital-based setting. Furthermore, this study revealed clinical scenarios of breast cancers with initial screening mammography: roughly three-tenths with breast symptoms, 5% with false negative results (IBC), 10% with true negative results (cancer detected at subsequent mammography) and 7% were attributed to non-adherence. Additional sonography might lower the incidence of IBC for women with initial screening mammography, especially for those with clinical symptoms. Further studies to evaluate the impact of repeated mammography and intervening sonography should be initiated to refine the role of mammography in modern health care system.

## Supplementary Information

**Additional file 1:** **Supplement table 1.** Overall survival discrepancy between Taiwanese breast cancers with initial diagnostic and screening mammography. **Supplement table 2.** BI-RADS distributions for women with the first screening mammography from 2012 to 2016. **Supplement table 3.** Time interval between screening mammography and cancer diagnosis among women with the initial screening mammography. **Supplement table 4.** Time interval between successive mammography examinations before cancer diagnosis among women with the initial screening mammography. MMG=mammography. **Supplement table 5.** Time interval between successive mammography examinations before cancer diagnosis among women with the initial screening mammography. MMG=mammography. **Supplement table 6.** Pathology offalse negativeresults and true negative resultsamong breast cancers with initial screening mammography.

## Data Availability

Cancer registry and administration data were anonymized by the Big Data Center, Taipei Veterans General Hospital (https://wd.vghtpe.gov.tw/bdc/Fpage.action?muid=11362&fid=10682), from where relevant data and materials may be available under reasonable request. Please contact corresponding author, Chi-Cheng, Huang if requesting the data. (chishenh74@gmail.com).
