# Supplementary material for: Characteristics of breast cancers detected by screening mammography in Taiwan: a single institute’s experience
Source: BMC Womens Health. 2023 Jun 21;23:330. doi: 10.1186/s12905-023-02445-6 (PMC10286479; doi:10.1186/s12905-023-02445-6)
Supplement: Supplementary file 1 — Additional file 1: Supplement table 1. Overall survival discrepancy between Taiwanese breast cancers with initial diagnostic and screening mammography. Supplement table 2. BI-RADS distributions for women with the first screening mammography from 2012 to 2016. Supplement table 3. Time interval between screening mammography and cancer diagnosis among women with the initial screening mammography. Supplement table 4. Time interval between successive mammography examinations before cancer diagnosis among women with the initial screening mammography. MMG=mammography. Supplement table 5. Time interval between successive mammography examinations before cancer diagnosis among women with the initial screening mammography. MMG=mammography. Supplement table 6. Pathology offalse negativeresults and true negative resultsamong breast cancers with initial screening mammography. [file 12905_2023_2445_MOESM1_ESM.docx]

Supplement table 1. Overall survival discrepancy between Taiwanese breast cancers with initial diagnostic and screening mammography.

| Initial mammography | Breast cancer case number | Case with mortality | Alive case | Overall survival rate |
| --- | --- | --- | --- | --- |
| Diagnostic | 600 | 47 | 553 | 92.17% |
| Screening | 291 | 9 | 282 | 96.91% |
| Total | 891 | 56 | 835 | 93.71% |

Supplement table 2. BI-RADS distributions for women with the first screening mammography from 2012 to 2016.

| BI-RADS | Final diagnosis of screening invitees | | | | | |
| --- | --- | --- | --- | --- | --- | --- |
|  | Breast cancer | | Benign condition | | Total | |
| 0 | 137 | 42.15% | 3425 | 13.3% | 3562 | 13.65% |
| 1 | 6 | 1.85% | 2951 | 11.45% | 2957 | 11.33% |
| 2 | 43 | 13.23% | 17797 | 69.03% | 17840 | 68.34% |
| 3 | 19 | 5.85% | 1408 | 5.46% | 1427 | 5.47% |
| 4 | 95 | 28.12% | 175 | 0.68% | 270 | 1.03% |
| 5 | 30 | 9.23% | 22 | 0.09% | 52 | 0.2% |
| Total | 325 |  | 25778 |  | 26103 |  |

Supplement table 3. Time interval between screening mammography and cancer diagnosis among women with the initial screening mammography (n=234 with one round of mammography).

| Total case number | | 79 | 5 | 29 | 6 | 85 | 30 | 234 |
| --- | --- | --- | --- | --- | --- | --- | --- | --- |
| Time interval (month) | 31 | 0 | 1 | 0 | 0 | 0 | 0 | 1 |
|  | 29 | 0 | 0 | 1 | 1 | 0 | 0 | 2 |
|  | 28 | 0 | 0 | 1 | 0 | 0 | 0 | 1 |
|  | 27 | 0 | 0 | 1 | 0 | 0 | 0 | 1 |
|  | 24 | 2 | 0 | 0 | 0 | 0 | 0 | 2 |
|  | 23 | 1 | 0 | 1 | 1 | 0 | 0 | 3 |
|  | 20 | 0 | 0 | 1 | 0 | 0 | 0 | 1 |
|  | 19 | 0 | 0 | 1 | 0 | 0 | 0 | 1 |
|  | 18 | 0 | 0 | 1 | 0 | 0 | 0 | 1 |
|  | 16 | 0 | 1 | 0 | 0 | 0 | 0 | 1 |
|  | 15 | 0 | 1 | 1 | 0 | 0 | 0 | 2 |
|  | 13 | 1 | 0 | 1 | 0 | 0 | 0 | 2 |
|  | 12 | 0 | 1 | 1 | 0 | 0 | 0 | 2 |
|  | 11 | 0 | 0 | 4 | 1 | 0 | 0 | 5 |
|  | 9 | 1 | 0 | 1 | 0 | 0 | 0 | 2 |
|  | 8 | 1 | 0 | 3 | 0 | 0 | 0 | 4 |
|  | 6 | 1 | 0 | 2 | 0 | 0 | 0 | 3 |
|  | 5 | 1 | 1 | 0 | 0 | 1 | 0 | 3 |
|  | 4 | 1 | 0 | 1 | 1 | 0 | 0 | 3 |
|  | 3 | 2 | 0 | 0 | 0 | 0 | 0 | 2 |
|  | 2 | 4 | 0 | 0 | 0 | 1 | 0 | 5 |
|  | 1 | 32 | 0 | 1 | 0 | 2 | 0 | 35 |
|  | <1 | 32 | 0 | 7 | 2 | 80 | 30 | 151 |
| BI-RADS | | 0 | 1 | 2 | 3 | 4 | 5 | Total case number |

Supplement table 4. Time interval between successive mammography examinations before cancer diagnosis among women with the initial screening mammography (n=72 with two rounds of mammography). MMG=mammography

| BI-RADS distributions | | Time interval (month) | | Case number(n) |
| --- | --- | --- | --- | --- |
| First MMG | Secondary MMG | First MMG | Secondary MMG |  |
| 0 | 0 | 3 | 2 | 1 |
| 0 | 0 | 1 | 1 | 1 |
| 0 | 0 | 29 | <1 | 1 |
| 0 | 2 | 1 | <1 | 1 |
| 0 | 2 | 3 | <1 | 1 |
| 0 | 3 | 4 | <1 | 1 |
| 0 | 3 | 9 | 8 | 1 |
| 0 | 3 | 20 | <1 | 1 |
| 0 | 4 | <4 | <3 | 35 |
| 0 | 4 | >24 | <1 | 3 |
| 0 | 4 | 15 | 0 | 1 |
| 0 | 4 | 24 | <1 | 1 |
| 1 | 4 | 1 | <1 | 1 |
| 1 | 4 | 16 | <1 | 1 |
| 2 | 0 | 6,12 | <1 | 2 |
| 2 | 0 | 23 | <1 | 1 |
| 2 | 2 | 14 | <1 | 1 |
| 2 | 2 | 24 | <1 | 1 |
| 2 | 4 | 16,17,20,23 | <1 | 4 |
| 3 | 0 | 13 | <1 | 1 |
| 3 | 2 | 32 | 9 | 1 |
| 3 | 3 | 7 | <1 | 1 |
| 3 | 4 | 13,15,23,25 | <1 | 4 |
| 3 | 5 | >24 | <1 | 2 |
| 4 | 3 | <1 | <1 | 1 |
| 4 | 4 | <1 | <1 | 2 |
| 4 | 4 | 7 | <1 | 1 |

Supplement table 5. Time interval between successive mammography examinations before cancer diagnosis among women with the initial screening mammography (n=19 with three rounds of mammography). MMG=mammography

| BI-RADS distributions | | | Time interval (month) | | | Case number(n) |
| --- | --- | --- | --- | --- | --- | --- |
| First MMG | Secondary MMG | Third MMG | First MMG | Secondary MMG | Third MMG |  |
| 0 | 0 | 0 | 18 | 1 | <1 | 1 |
| 0 | 0 | 4 | 11 | 12 | <1 | 1 |
| 0 | 0 | 5 | 12 | <1 | <1 | 1 |
| 0 | 3 | 2 | 1 | 12 | 11 | 1 |
| 0 | 3 | 4 | 21 | 21 | <1 | 1 |
| 0 | 4 | 4 | 6,6,7 | <1 | <1 | 3 |
| 2 | 0 | 4 | 25 | 1 | <1 | 1 |
| 2 | 0 | 4 | 26 | 3 | <1 | 1 |
| 2 | 2 | 4 | 22 | 10 | <1 | 1 |
| 2 | 4 | 4 | 23,24 | <1 | <1 | 2 |
| 3 | 0 | 4 | 21,26 | <1 | <1 | 2 |
| 3 | 0 | 3 | 9 | 1 | <1 | 1 |
| 3 | 3 | 4 | 6 | 6 | <1 | 1 |
| 4 | 0 | 4 | 13 | 3 | 3 | 1 |
| 4 | 4 | 4 | <1 | <1 | <1 | 1 |

Supplement table 6. Pathology of (a) false negative (interval cancer) results and (b) true negative results (diagnosed from scheduled subsequent mammography) among breast cancers with initial screening mammography.

(a)

| ER | PR | HER2 | Ki-67 | grade | Pathological stage | Histology |
| --- | --- | --- | --- | --- | --- | --- |
| 0 | 0 | +1 | 90 | G3 | T1cN0 | IDC |
| 90 | 70 | +1 | 10 | G2 | T2N1 | IDC |
| 90 | 85 | +1 | NA | G2 | 2.2cm | DCIS |
| 80 | 90 | +2 | 30 | G2 | T1bN0 | IDC |
| 80 | 60 | +1 | 15 | G2 | T1bN0 | IDC |
| 90 | 10 | +1 | 30 | G2 | T1bN0 | IDC |
| 0 | 0 | 2+ | 30 | G3 | T2N1 | IDC |
| 0 | 0 | 3+ | 34 | G2 | T2N1 | IDC |
| 0 | 0 | +2 | 40 | G3 | T2N1 | IDC |
| 0 | 0 | +3 | 30 | G2 | T1N0 | IDC |
| 0 | 0 | +3 | 75 | G2 | T2N0 | IDC |
| 90 | 30 | +1 | 10 | G2 | T1N0 | IDC |
| 0 | 0 | +3 | 25 | G3 | T1N0 | IDC |
| 30 | 0 | 0 | 12 | G2 | T2N0 | ILC |
| 90 | 90 | 0 | 14 | G1 | T2N0 | Mucinous |
| 90 | 90 | +1 | 5 | G2 | T1N0 | IDC |
| 90 | 90 | 0 | 14 | G1 | T2N0 | Mucinous |

(b)

| ER | PR | HER-2 | KI | grade | Stage | pathology |
| --- | --- | --- | --- | --- | --- | --- |
| 90 | 90 | +1 | 7 | G2 | T1aN0 | IDC |
| 80 | 80 | 0 | 20 | G2 | T1cN0 | IDC |
| 90 | 90 | +1 | 20 | G3 | T1Nm | IDC |
| 90 | 50 | +1 | 15 | G2 | T1aN0 | IDC |
| 90 | 80 | +1 | 2 | G1 | T1aN0 | IDC |
| 90 | 90 | +1 | 20 | G2 | T1aN0 | IDC |
| 90 | 90 | +1 | 25 | G2 | T1bN0 | IDC |
| 90 | 40 | +1 | NA | G2 | T2N0 | MicroIDC |
| 70 | 80 | +1 | NA | G1 | 0.2cm | Papillary Ca |
| 95 | 95 | +1 | NA | G1 | 0.2cm | DCIS |
| 0 | 0 | +1 | NA | G2 | 0.8cm | DCIS |
| 95 | 95 | +1 | 9 | G1 | T1N0 | IDC |
| 95 | 70 | +2 | 30 | G3 | NA | IDC |
| 40 | 0 | +3 | NA | G2 | 0.6cm | DCIS |
| 95 | 95 | +1 | 15 | G2 | T1bN0 | IDC |
| 5 | 2 | +1 | NA | NA | 0.5cm | DCIS |
| 90 | 90 | +1 | - NA | NA | 0.3cm | DCIS |
| 80 | 30 | +1 | 5 | G2 | T2N0 | IDC |
| 95 | 45 | +1 | 2 | G2 | T1bN0 | IDC |
| 90 | 50 | 0 | 5 | G2 | T2N0 | IDC |
| 0 | 0 | +2 | NA | G3 | 0.4cm | DCIS |
| 90 | 90 | +1 | NA | NA | 0.3cm | DCIS |
| 90 | 90 | +1 | 10 | G1 | T1 | DCIS |
| 90 | 25 | +2 | NA | NA | 0.5cm | DCIS |
| 90 | 90 | +1 | NA | NA | PTis | DCIS |
| 0 | 0 | +3 | NA | G3 | 0.4cm | DCIS |
| 0 | 0 | +3 | 80 | G2 | T1N0 | IDC |
| 80 | 50 | 0 | NA | G2 | 2cm | DCIS |
| 80 | 50 | 0 | NA | G2 | 2cm | DCIS |
| 90 | 90 | +1 | 7 | G2 | T1aN0 | IDC |
| 95 | 95 | +1 | NA | NA | Tis | DCIS |
| 0 | 0 | +3 | NA - | NA | Tis | DCIS |
| NA | NA | NA | NA | NA | NA | Microinvasive ductal carcinoma |
| 90 | 50 | 0 | 5 | G2 | T1cN0 | Mucinous carcinoma |
